# Application of machine learning in predicting survival outcomes involving real-world data: a scoping review

**DOI:** 10.1186/s12874-023-02078-1

**Published:** 2023-11-13

**Authors:** Yinan Huang, Jieni Li, Mai Li, Rajender R. Aparasu

**Affiliations:** 1 Department of Pharmacy Administration, School of Pharmacy, University of Mississippi, University, MS 38677 USA; 2 Department of Pharmaceutical Health Outcomes and Policy, College of Pharmacy, University of Houston, Houston, TX 77204 USA; 3 Department of Industrial Engineering, Cullen College of Engineering, University of Houston, Houston, TX USA

**Keywords:** Machine learning, Real-world datasets, Random survival forest, Neural network

## Abstract

**Background:**

Despite the interest in machine learning (ML) algorithms for analyzing real-world data (RWD) in healthcare, the use of ML in predicting time-to-event data, a common scenario in clinical practice, is less explored. ML models are capable of algorithmically learning from large, complex datasets and can offer advantages in predicting time-to-event data. We reviewed the recent applications of ML for survival analysis using RWD in healthcare.

**Methods:**

PUBMED and EMBASE were searched from database inception through March 2023 to identify peer-reviewed English-language studies of ML models for predicting time-to-event outcomes using the RWD. Two reviewers extracted information on the data source, patient population, survival outcome, ML algorithms, and the Area Under the Curve (AUC).

**Results:**

Of 257 citations, 28 publications were included. Random survival forests (*N* = 16, 57%) and neural networks (*N* = 11, 39%) were the most popular ML algorithms. There was variability across AUC for these ML models (median 0.789, range 0.6–0.950). ML algorithms were predominately considered for predicting overall survival in oncology (*N* = 12, 43%). ML survival models were often used to predict disease prognosis or clinical events (*N* = 27, 96%) in the oncology, while less were used for treatment outcomes (*N* = 1, 4%).

**Conclusions:**

The ML algorithms, random survival forests and neural networks, are mainly used for RWD to predict survival outcomes such as disease prognosis or clinical events in the oncology. This review shows that more opportunities remain to apply these ML algorithms to inform treatment decision-making in clinical practice. More methodological work is also needed to ensure the utility and applicability of ML models in survival outcomes.

**Supplementary Information:**

The online version contains supplementary material available at 10.1186/s12874-023-02078-1.

## Background

Survival analysis or time-to-event analysis has gained interest in health service research, as predicting the time to an outcome of interest is critically important in clinical research [1, 2]. Survival analysis refers to a group of statistical methods designed to handle time-to-event (TTE) outcome prediction. A challenge in the context of time-to-event data is that while survival times for some subjects will be known as they have experienced the event during the study period, but for a subset of the group, they may not have yet experienced the event during the study period; therefore, their survival time will still be unknown. This phenomenon, often known as censoring, may happen due to a variety of reasons, such as patients have not yet developed the relevant outcome, such as disease progression or death by the end of the study period; the study subjects can also be lost to follow-up during the study, or the patients experience another event that prohibits the further follow-up. Survival analysis must account for the censoring to obtain valid estimates for inferences. Survival analysis is particularly important in clinical oncology research as most oncology studies involve the assessment of time-to-event outcomes, including evaluating a patient’s overall survival (OS) and progression-free survival (PFS) after a cancer diagnosis or disease recurrence [3, 4]. Traditionally, the Cox Proportional Hazards (CPH) model, as a semi-parametric model, is the most widely applied approach to overcome the issue of censoring for the analysis of time-to-event data [5–7]. However, the CPH model has several limitations: reliance on the statistical assumption and not being tailored to high-dimensional complex data.

Machine learning (ML), a branch of artificial intelligence, is a family of data analytical methods that enables the capture of patterns behind complex data [8, 9] and has gradually become a popular approach for risk prediction in the healthcare research [10]. With rapid generation and availability of real-world data (RWD) in the medical field, ML techniques have played an important role in using complex and large RWD to provide evidence in clinical research and practice, including clinical disease diagnosis, treatment outcomes, and disease progression [11–13]. In the health service areas, ML methods, including random forests (RF), k-nearest neighbors (KNNs), support vector machines (SVMs), and neural networks (NNs), are common methods [10]. Empirical evidence has shown that various ML methods have been adjusted to analyze time-to-event data. For example, Moncada-Torres et al. used Netherlands Cancer Registry data involving 36,658 breast cancer patients to compare three ML models (random survival forest, SVM, and extreme gradient boosting) versus traditional regression-based CPH in survival outcomes [14]. Findings showed that ML models effectively obtained area under the receiver operating characteristic (AUROC) of 0.63 comparable to classical CPH. [14] Another study analyzed Alberta’s electronic health record data for the development of five ML models (penalized regression Ridge, least absolute shrinkage and selection operator [LASSO], elastic net, random survival forest, and gradient boosting) to predict time to incident hypertension in a Canadian population and demonstrated similar performance (AUC 0.76–0.78) between these ML models versus traditional CPH [15]. Despite many advances in ML methods and the growing need for time-to-event analysis, there is a gap in systematic understanding of the application of ML methods for time-to-event analyses.

Over the years, many ML-based approaches have been developed to diagnose diseases, predict disease severity prognosis, estimate probabilities of hospital readmissions, etc [16–18]. As the growth of interest in time-to-event outcomes, the use of ML solutions for predicting survival outcomes are being proposed, e.g., for early detection of dementia disease or for estimating the development of oral cancer [19, 20]. As far as we are aware, no reviews exist specifically involving studies of ML models to predict time-to-event outcomes from real-world structured data. Therefore, to fill this evidence gap, we conducted this review of ML methods used for survival prediction using the RWD in healthcare. This review aims to characterize (1) the common ML methods that have been utilized for survival prediction involving RWD; (2) the performance of these ML models along with the data source, study design, sample size, and validation approaches; (3) the diseases and the type of time-to-event outcomes; and (4) the quality of these models. This review serves as a primer for future research in developing novel ML-based predictive algorithms in survival prediction.

## Methods

This scoping review utilized the Preferred Reporting Items for Systematic Reviews and Meta-Analyses (PRISMA-ScR) to achieve the study aims and to characterize ML studies on time-to-event outcomes using the RWD [21]. To guide data extraction for ML prediction models, two checklists, including the Critical Appraisal and Data Extraction for Systematic Reviews of Prediction Modeling Studies (CHARMS) checklist and Machine Learning Methods in Health Economics and Outcomes Research Checklist were utilized  [22, 23].

### Databases search and search strategy

This scoping review searched PUBMED and EMBASE online databases from database inception through March 2023. Relevant studies involving ML methods for survival analyses based on real-world datasets were included. With guidance from the librarian for the Health Sciences, the author team developed search strategies. For these database searches, the search strategy included search terms involving “machine learning,” “survival outcome,” and “real-world database.” The search syntax related to ‘real-world database” was defined based on US FDA; according to the US FDA, the RWD in the healthcare field refers to the data relevant to population health status or the delivery of healthcare, and such RWD can be collected from multiple sources: (1) claims and billing activities, (2) electronic health records (EHRs), (3) disease registries, e-health services, and other wearable technology-driven services. For a focused scoping review, searches were limited to non-wearable real-world data. In addition, the survival outcome refers to the time-to-event outcome; syntax related to this term was developed based on prior literature. All identified citations were imported into an electronic Excel sheet. The details of search strategies and results as per each database are shown in Additional Supporting File 1: Part I, Full Search Strategy.

### Eligibility criteria and study selection

Citations from all databases were imported into the Excel sheets. After removing duplicates, the unique articles were imported into Excel sheets for titles and abstract review. Authors (YH, JL, ML) together performed titles and abstracts review and conducted the screening. Any conflict was solved through a discussion involving a fourth author (RR).

For full article eligibility screening, articles available in the complete paper were retrieved. Studies were deemed eligible if they used ML methods for survival analyses based on real-world non-wearable data. We included only ML-based survival prediction using real-world datasets, including patient charts or registries, administrative claims data, and electronic health records. We excluded studies with the following characteristics: (1) no population-level structured data (e.g., randomized controlled trial (RCT), simulation data, imaging data); (2) without ML-based modeling (e.g., use ML for feature selection only, or just involve statistical learning methods); (3) no survival prediction (e.g., binary classification of survival, no time-to-event outcome), (4) primary research only (e.g., literature reviews excluded). Details of inclusion/exclusion criteria are also provided in Additional Supporting File 1: Part II. Inclusion/Exclusion Criteria for Screening Articles.

### Data extraction and synthesis

Three authors performed data extraction using a standardized form based on Microsoft Excel spreadsheets. This study extracted information was as follows: (1) Characteristics of studies, including first author and publication year, data source, study population and setting, sample size, survival outcome predicted (see Additional supporting file 2: Supporting information Table S1); (2) Characteristics of ML models, including ML algorithms used, model validation, ML model performance (see Additional supporting file 2: Supporting information Table S2); and (3) Quality assessment (see Additional supporting file 2: Supporting information Table S3). Specifically, the area under the curve (AUC) was extracted as the evaluation metric because the AUC has the advantage of providing a comprehensive summary of the models’ predictive ability. These supporting documents were organized to facilitate linkage across studies. Due to variations in the study design and ML algorithm applied and heterogeneity in statistical analysis, the quantitative summary of studies was not feasible, and hence, all included studies were summarized qualitatively. The AUC with a 95% confidence interval (if available) was extracted to describe model performance. To assist in presenting AUC by ML methods in data synthesis, we selected the ML model with the maximum AUC if more than one ML model was developed based on the same ML algorithm. In addition, the AUC values for validation datasets were given the priority. We visually presented the extracted data using boxplots and beeswarm plots, categorized by the type of ML algorithms. These plots were generated using the "beeswarm" package in R software [24]. Further, to allow a high-level comparison of ML studies, these studies were then grouped into two categories: ML-only studies and studies involving comparison of ML versus CPH. To characterize those comparative studies involving ML versus CPH, two authors independently abstracted both the performance of ML models and CPH. We also extracted findings related to the relative performance of the ML over the CPH in the prediction of survival analyses.

### Quality assessment

The prognosis study quality tool and clinical decision rule assessment tool (QUIPS) were used to assess the methodological aspects and quality of the included studies critically [25]. The QUIPS focused on assessing the following elements: study cohort definition, adequacy of prognostic factor measurement, outcome variable measurement, confounding adjustment, and statistical analysis reporting. For each domain, the ratings include ‘yes,’ ‘partly,’ and ‘no.’ The overall strength of evidence for each study was based on grading the above six domains. If all domains were designated ‘yes’ for high ratings, then the overall quality of articles was good. If at least one domain was designated ‘partly,’ then the overall quality of the evidence was fair. If one or more domains were designated ‘no,’ the overall strength of evidence was poor. See Additional supporting file 2: Supporting information Table S3 for quality assessment results.

## Results

**Fig. 1:**
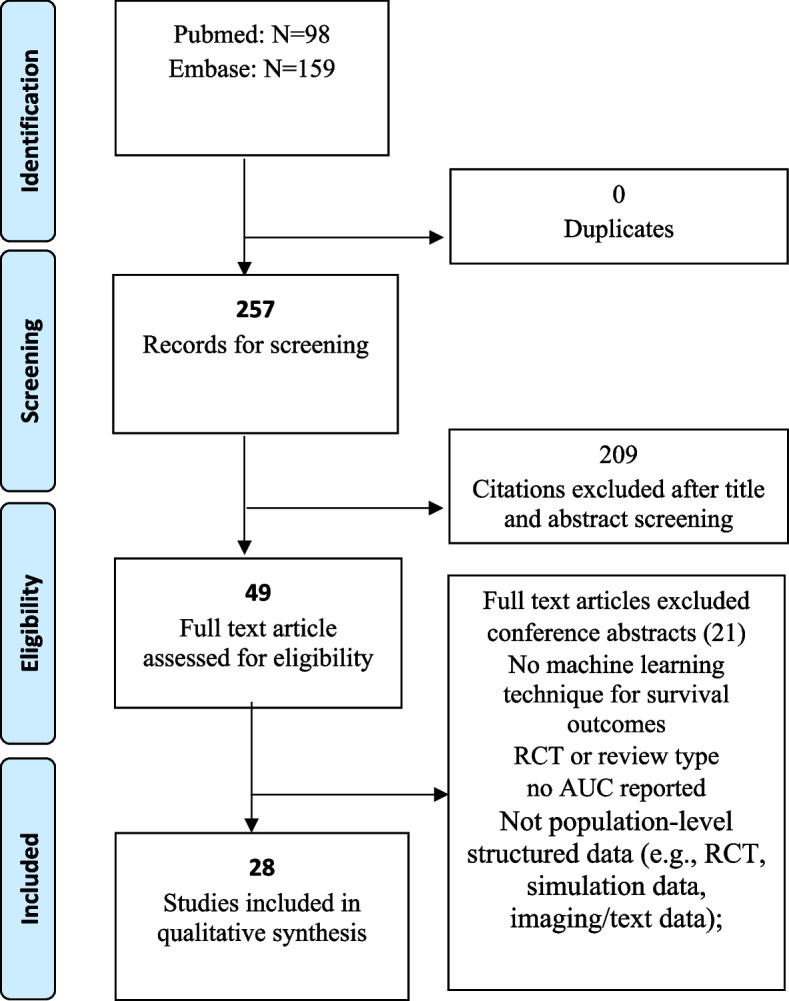
Flow diagram for study selection

This scoping review search identified a total of 98 studies from the PubMed and 159 studies from the Embase. After duplication elimination and abstract and title screening, studies were considered potentially relevant and selected for full-article review. Among these, 28 peer-reviewed studies involving at least one unique ML model across a broad list of patient populations and settings were included in this review (Fig. 1).

### Study characteristics

#### Data source and sample size

The majority of these studies (*N*= 14) were conducted using data from the US setting [26–39]. Among these US studies, most of them used administrative claims datasets [26, 27, 30, 31, 36, 39] (*N* = 6), including SEER-Medicare, Veteran health administrative claims, followed by electronic health records or electronic medical records [32–34, 37, 38] (*N* = 5), and a few used patient registry cohort datasets [28, 29, 35] (*N* = 3). The remaining non-US studies used datasets from Europe [40–45] (*N* = 6), including Italy, Netherlands, Denmark, Switzerland, or Germany, and a few others used data from England (*N* = 3), China (*N* = 4), or India (*N* = 1). The median sample size was 10,614 (range: 142- 247,960 patients).

#### Study population and time-to-event outcomes

Most of these studies involving ML-based prediction for survival analyses focused on cancer patients [26, 27, 30, 31, 34, 36, 38, 39, 42, 43, 46, 47] (*N* = 12 studies); for ML studies in oncology, these models were used to predict their survival outcomes or cancer recurrence.

The remaining studies focused on patient populations in the cardiology [28, 35, 48, 49], COVID-19 [37, 50, 51], diabetes [29, 40, 41, 45], schizophrenia disorder patients [52], HBV infection [53], inpatients patients [32], those undergoing heart transplantation [33], or intensive care unit (ICU) patients [54]. Across these non-cancer disease areas, these ML studies predicted clinical outcomes, such as the development of cardiovascular events [29, 40, 41, 45], the incidence of sudden cardiac arrest or venous thromboembolism or ventricular fibrillation, and death. Only one study used ML for treatment outcomes [52]. A detailed summary of included studies is provided in e-supporting Table 1.

**Table 1:** ML algorithms used in the studies and featuring studies (*N* = 28 studies)

**Type of ML Algorithms**	**Number of Studies**^d^	**Featuring Studies**
**Tree-based Methods**		
Random survival forests	16	26–28,31–34,36,42,43,45–49,53
Boosted tree methods^a^	7	31,34,42,43,45,51,53
**Neural Networks**		
Artificial neural networks^b^	11	30,31,37,39–41,43,44,46,47,49,50
**Support Vector Machine**	4	34,35,42,53
**Regularization**^c^	4	
**Other algorithms**		
Naives bayes	3	29,35,53
K-Nearest Neighbors	1	35
Multi-layer Perceptron	1	34

*ML* Machine learning, *LASSO* Least absolute shrinkage and selection operator, *NN* Neural networks, *CNN* Convolutional neural network, *RNN* Recurrent neural network, *DL* Deep learning, KNN The k-nearest neighbors

^a^includes ada-boost, gradient boosting, gradient descent boosting, boosting, XGBoost

^b^includes CNN, RNN, DNN, deep stacking networks, and ensemble of DL methods

^c^includes LASSO (L1 regularization), Ridge Regression (L2 regularization), or Elastic-Net

^d^Since most studies have applied more than 1 machine learning algorithms, therefore the sum of the number of studies by machine learning method is greater than included studies (*N* = 28)

### Characteristics of ML Models

#### Use of ML for survival outcomes

The types of ML algorithms used are reported in Table 1. From this review, the popular ML algorithms for survival analyses include random survival forests (*N* = 16) [26–28, 31–34, 36, 42, 43, 45–49, 53], boosted tree methods [31, 34, 42, 43, 45, 51, 53], and artificial neural networks [30, 31, 37, 39–41, 43, 44, 46, 47, 49, 50]. Support vector methods [34, 35, 42, 53] and regularization (LASSO, ridge, elastic net) [43, 49, 52, 53] were also common, and other algorithms included naïve bayes [29, 35, 53], K-nearest neighbor [35], multi-layer perceptron [34]. Table 2 provides a description of these ML algorithms.

**Table 2:** Description of ML methods

Method	Basic Concept	How It Works	Pros	Cons
Random Survival Forest	An ensemble tree-based learning algorithm specialized for survival analysis	Trains multiple decision trees on different subsets of the data and averages predictions. Time-to-event data is used to split nodes and generate survival curves	Handles large, high-dimensional datasets; automatically handles feature interactions; robust to outliers	Can be slow on large datasets; may overfit without careful tuning
Boosted Tree	An ensemble tree-based method that combines weak predictors to form a strong predictor	Trains simple models in a sequential manner. Each new tree tries to correct the mistakes of the previous one	Can handle different types of data; reduces bias and variance; highly accurate	Can overfit if too many trees are used; requires careful tuning; less interpretable
Artificial Neural Network	A model inspired by the human brain, with layers of interconnected nodes or "neurons"	Each neuron receives input from previous neurons, applies a transformation, and sends the output to next neurons. Learning involves updating the transformation parameters	Can model complex nonlinear relationships; highly flexible and adaptable	Requires lots of data and computational resources; hard to interpret; prone to overfitting
Support Vector Machine	A binary classification method that finds the hyperplane maximizing the margin between classes	Finds the hyperplane that maximizes the distance between closest points of different classes. Can use kernels for nonlinear boundaries	Effective in high dimensional spaces; robust to overfitting in the right dimensional space	Not suitable for larger datasets; requires careful choice of kernel; not directly applicable for multi-class problems
Regularization (LASSO, Ridge)	Linear models with added terms in the loss function to prevent overfitting	LASSO (L1 regularization) and Ridge (L2 regularization) add penalty terms to the loss function that shrink coefficients towards zero	Prevents overfitting; reduces model complexity	May lead to underfitting if regularization parameter is not tuned correctly
K-Nearest Neighbor	A simple algorithm that predicts based on the k closest training examples	For a new instance, finds the k nearest instances in the training set and predicts based on their output	Simple to understand and implement; no assumptions about data distribution	Computationally expensive for large datasets; sensitive to irrelevant features; performance depends on the choice of k
Multi-Layer Perceptron	A type of artificial neural network with one or more hidden layers	Works as a simple neural network with added hidden layers for complex transformations	Can model complex nonlinear relationships; flexible and adaptable	Requires lots of data and computational resources; hard to interpret; prone to overfitting
Naive Bayes	Probabilistic classifier based on Bayes' theorem with strong (naive) independence assumptions between features	Each feature independently contributes to the probability of the class. Class with the highest probability is chosen	Fast and efficient; performs well with high dimensions; requires less training data	Assumes feature independence which is often not the case; can be biased if a class lacks representation in the training data

#### ML model performance

**Fig. 2:**
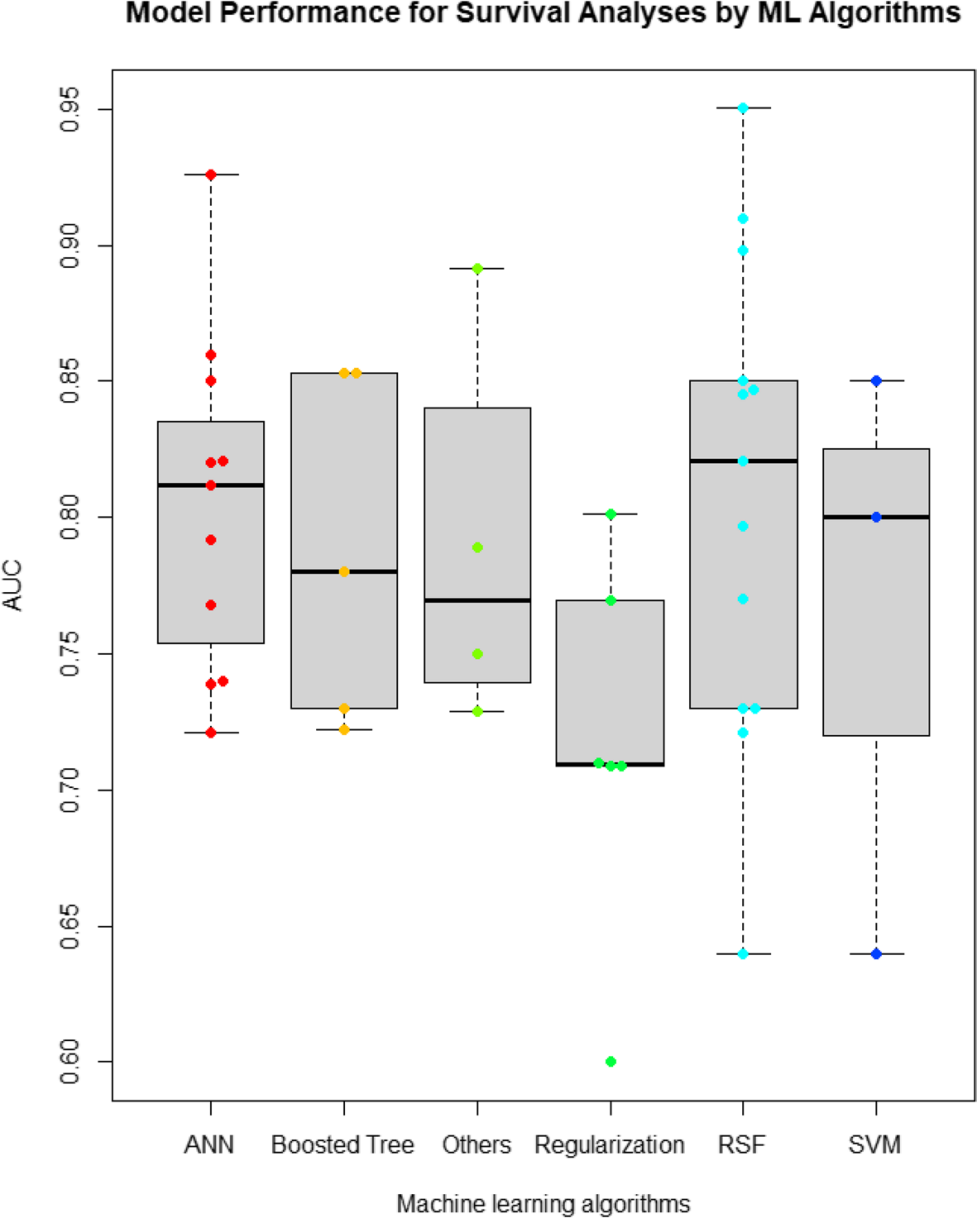
ML Performances for survival analyses

Across these studies, while three studies [28, 33, 45] failed to report model performance in AUC, others reported AUC for model evaluation. Among those studies reporting AUC for evaluation of model performance, there was a variation across the AUCs reported, with their mean at 0.7852 and their median at 0.789 (IQR: 0.73–0.847; range: 0.6–0.9503). While one study developed one ML model [52] with an AUC below 0.7, most of these studies developed at least one ML model with an AUC above 0.70. The boxplot and beeswarm plot of model performance based on the AUC, stratified by the type of ML algorithms, are shown in Fig. 2. The descriptive findings of the AUC across these ML models are shown in Table 3.

**Table 3:** Descriptive statistics of AUC by ML algorithms

**ML category**	Number of models^d^	Mean (STD)	Median	Min	Max	IQR
Random survival forests	13	0.8084	0.821	0.64	0.9503	0.73–0.85
Boosted tree methods^a^	5	0.7876	0.78	0.722	0.853	0.73–0.853
Artificial neural networks	11	0.7999	0.802	0.721	0.926	0.747–0.8208
Support Vector Machine	3	0.7633	0.8	0.64	0.85	0.72–0.825
Regularization^b^	6	0.7164	0.7095	0.6	0.801	0.709–0.7546
Other algorithms^c^	4	0.7899	0.7695	0.7287	0.8917	0.7447–0.8147

*Abbreviations*: *ML* Machine learning, *NNs* Neural networks, *RF* Random forest, *DT* Decision tree, *SVM* Support vector machine, *STD* Standard deviation, *IQR* The interquartile range.

^a^It includes adaboost, gradient boosting, gradient descent boosting, boosting, XGBoost

^b^It includes Lasso (L1 regularization), ridge regression (L2 regularization), and elastic-net algorithms

^c^It includes: naives bayes, KNN or MLP

^d^the total number of studies may differ from than total number of included studies, because some studies used more than 1 ML algorithms and also models with no AUC reported were excluded

#### Model validations

Among all included studies, twenty-five studies (89%) applied model validation. Table 4 details model validation methods among these included studies. Nineteen studies used internal validation, with fifteen studies randomly split datasets into a training set and a test set for validation of model performance [26, 27, 29, 31, 32, 36, 38–41, 44, 46, 49, 50, 53], while four studies internally validated model performance using cross-validation methods [35, 42, 48, 52]. Six studies applied external validation methods, including using an independent dataset for model performance validation [30, 34, 37, 43, 47], or used prospective validation [51]. Still, three studies did not report any validation methods [28, 33, 45].

**Table 4:** Overview Of methods for model validation across studies (*N* = 28 studies)

Type of validation methods	Number of studies	Featuring studies
**Internal validation**	19	
Training/testing split	15	26,27,29,31,32,36,38–41,44,46,49,50,53
Resampling involving k-fold cross-validation	4	35,42,48,52
**External validation**	6	
An independent dataset for validation	5	30,34,37,43,47
Prospective validation	1	51
**No validation**	3	28,33,45

#### Comparison between model performance of ML vs. CPH

A total of 17 studies (61%) compared the performance of ML models with the traditional regression-based CPH. Most studies (*N*= 15 studies, 88%) reported that ML had better performance than CPH models [26, 30–32, 34, 36, 38–43, 48–50]. Only one study reported that ML algorithms did not surpass the CPH model [27], and one study did not make a comparison, although it included CPH [29]. Details can be found in e-supporting Table 1.

#### Quality assessment

Among the included studies, a majority had high quality based on the appraisal of six domains of the QUIPS tool. Details of quality assessment for all included studies are summarized in e-supporting doc Table 3.

## Discussion

This is the first scoping review that specifically evaluated the application of ML in survival analyses based on 28 studies utilizing RWD. This scoping review summarized ML-based studies for survival prediction involving RWD in observational studies. This review also provides the utility of these ML methods for survival analyses using RWD.

### ML methods common in survival prediction and their model performance

The existing literature appling ML approaches in survival risk prediction is limited, and this scoping review found random survival forests and neural networks as popular ML algorithms for survival outcome prediction. As a nonparametric tree-based ensemble method, a random survival forest is an extension of a random forest and is suitable for the analysis of censored time-to-event outcomes for dynamic prediction [55, 56]. Several recent studies applied random survival forest for analyzing time-to-event data to predict survival in cardiology or oncology patients [57, 58]. Neural network is also a popular approach for survival prediction, e.g., for cancer survival prediction [59]. Only a few studies identified in this review combined different ML modeling approaches. As a best practice, future studies should utilized combined ML approaches as an ML-based modeling strategy.

This review additionally offers several insights into the development of ML models for survival risk prediction. Firstly, these models utilizing RWD are limited by the quality of underlying training datasets. As such, to obtain reliable models, a high-quality healthcare dataset that contains a large enough sample and suitable quality with rich variables of predictive value is required for the development of ML models [60, 61]. In this scoping review, the underlying real-world data for ML model development often involves electronic medical records. The quality of underlying RWDs for ML training is very important. In particular, the underlying databases should contain variables or information fully reflective of prognostic and predictive value. Continued efforts to link different sources of data will strengthen the application of ML for survival applications to generate real-world evidence. Furthermore, most studies used internal validation, and only a few studies used external validation. In another systematic review, Brnabic et al. summarized common ML methods used for real-world clinical decision-making, and they also found that only two studies performed external validation out of 34 publications [62]. There is a strong need to employ both internal and external validation approaches for high-quality ML models. Also, model evaluation of an ML model performance is suggested involving a prospective dataset. Similar to the need for high-quality datasets for ML algorithm development, external validation using another independent or prospective dataset is critical for successfully translating ML models into clinical applications. Practical guides and good modelling practice recommendations for the application of ML methods based on RWD need to be developed.

### Comparative performance between ML and CPH for survival prediction

This current review also demonstrates that compared to conventional CPH, most ML models achieved better performance in the context of complex, high-dimension datasets, adding to a body of literature about comparing ML with traditional models [66, 67]. Several systematic reviews compared ML and traditional logistic regression for binary outcome prediction, showing ML algorithms, such as random forest, gradient boosting, and neural networks, significantly outperformed logistic regression [66, 67]. However, there is a lack of insights into the comparative performance of ML versus conventional CPH in the context of survival outcomes. This review adds insights into the comparison of ML and CPH for survival prediction and shows the improved performance of the ML model over  CPH in the context of the time-to-event outcome. The conventional Cox model is not intended to deal with complex datasets with high dimensionality and a large number of features; instead, they are more adept at a subset of predictors. For example, it is suggested that using feature reduction methods, e.g., penalty-based LASSO (L1), ridge regularization, or elastic-net regularization, and then modeling using the Cox regression methods could improve the performance of CPH [63–65]. Overall, a head-to-head meta-analysis comparing ML models and classical CPH in the context of survival analyses is needed.

Furthermore, the intent of this review is not to clarify the most superior ML algorithm for survival prediction. Instead, the selection of the most suitable ML algorithm for survival analyses should be based on the particular research question as well as the characteristics of underlying datasets, e.g., how large the sample size is, how many variables are available, and how balanced the datasets are. For instance, if the population size is not large enough, the use of neural networks may result in an overfitting problem, while the SVM approach is advantageous for dimensionality reduction but requires careful tuning of the kernel number.

### Future of ML-based survival models using RWD

Although ML approaches are increasingly used for survival prediction, they have been mostly used for predicting future clinical events in oncology areas. There remain opportunities for future studies in other disease states to address the prediction of clinical events in other diseases. This review found ML survival models were often used to predict disease prognosis or clinical events. There is a need to use these ML-based survival methods to address treatment-related events such as dose titration, discontinuation, and switching doses. More methodological work is also needed to address the relative performance of ML approaches with traditional CPH. Furthermore, validation of ML models in external validation cohorts could improve the utility of these models. However, almost all studies in our review only used simple internal validation. Future studies in the application of ML in survival outcomes might improve by making ML algorithms externally validated across various health settings to facilitate its clinical utility.

### Limitations

We also acknowledge some limitations. First, this study provides the value of ML approaches for survival analyses using RWD in healthcare. However, this information may not be sufficient to select an ML for survival analyses due to the diversity of clinical outcomes assessed and the variety of datasets used among these studies. A more detailed assessment of model performance across these types of ML approaches under specific clinical outcomes can provide the suitability of ML for improved prediction. Second, another valuable emphasis would consider the comparison of ML with traditional regression-based CPH. In addition, this study also has limitations in terms of methodologic exclusion. As the ML algorithms used for survival analyses are based on observational studies, we only included works that are developed in real-world non-wearable datasets. We acknowledge that some studies were excluded due to their use of RCT data or wearable datasets. Lastly, although calibration provides information on agreement between the observed outcomes and the values predicted by the models, calibration could not be quantitatively presented due to limited studies reporting calibration statistics.

## Conclusions

This is the first scoping review that specifically focused on applying ML in time-to-event outcomes using RWD in healthcare. This scoping review found random survival forests and neural networks as the most popular ML methods for survival prediction using RWD, predominantly in oncology. These ML survival models were mainly used to predict disease prognosis or clinical events. This review found variations in the reported performance across multiple ML approaches with a mean AUC of 0.78 and a median of 0.79. Future studies could consider focusing on the application of ML in survival outcome prediction in other disease areas. There remain opportunities to apply these ML algorithms for survival prediction of the treatment outcomes that can inform clinicians about treatment decision-making. More methodological work is also needed, especially external validation and comparative performance, to ensure the utility and applicability of these ML models in survival outcomes.

### Supplementary Information

**Additional file 1. **Part I, Full Search Strategy. Part II. Inclusion/Exclusion Criteria for Screening Articles.

**Additional file 2: Table S1. **Characteristics of Included Studies on ML Predictive Models for Survival Analyses (*N*=28 studies). **Table S2. **Performances of Included Studies on Survival Analyses Using ML Algorithms (*N*=28 Studies). **Table S3. **Risk of Bias Assessment (*N*=28 studies).

**Additional file 3. **Preferred Reporting Items for Systematic reviews and Meta-Analyses extension for Scoping Reviews (PRISMA-ScR) Checklist.

**Additional file 4.**

## Data Availability

The corresponding author can provide the material used and data analyzed on request.

## Abbreviations

CMS: Centers for Medicare and Medicaid Services
ML: Machine learning
NN: Neural networks
AUC: Area Under the Curve
RCT: Randomized controlled trial
PRISMA-ScR: Preferred Reporting Items for Systematic Reviews and Meta-Analysis Extension for Scoping Reviews
CHARMS: CHecklist for critical Appraisal and data extraction for systematic Reviews of prediction Modelling Studies
QUIPS: The Quality in Prognosis Studies
IQR: Interquartile range
RSF: Random survival forest
SVM: Support vector machine
KNN: K-nearest neighbor algorithm
EHR: Electronic health record
DT: Decision tree
ROB: Risk of bias
DL: Deep learning
