# Supplementary material for: Application of machine learning in predicting survival outcomes involving real-world data: a scoping review
Source: BMC Med Res Methodol. 2023 Nov 13;23:268. doi: 10.1186/s12874-023-02078-1 (PMC10641971; doi:10.1186/s12874-023-02078-1)
Supplement: Supplementary file 1 — Additional file 1. Part I, Full Search Strategy. Part II. Inclusion/Exclusion Criteria for Screening Articles. [file 12874_2023_2078_MOESM1_ESM.docx]

**Additional Supporting File 1: Part 1, Full Search Strategy**

**Databases Searched**

| Date | Name | Records |
| --- | --- | --- |
| 3/9/2023 | PUBMED | 98 |
| 3/9/2023 | EMBASE Ovid | 159 |

| **Database: PUBMED Search Date: March 9th 2023** | | |
| --- | --- | --- |
| 1 | machine learning[Title/Abstract] | 81,654 |
| 2 | machine learning[MeSH Terms] | 54,622 |
| 3 | 1 OR 2 | 106,016 |
| 4 | electronic health records[Title/Abstract] OR electronic health record[Title/Abstract] OR electronic medical record[Title/Abstract] OR electronic medical records[Title/Abstract] OR claims data[Title/Abstract] OR claims dataset[Title/Abstract] OR claims datasets[Title/Abstract] OR administrative claim[Title/Abstract] OR administrative claims[Title/Abstract] OR disease registries[Title/Abstract] OR disease registry[Title/Abstract] OR registry data[Title/Abstract] OR registry dataset[Title/Abstract] OR registry datasets[Title/Abstract] | 76,851 |
| 5 | ((((electronic medical record[MeSH Terms]) OR (electronic medical records[MeSH Terms])) OR (claims analyses[MeSH Terms])) OR (analysis, claims[MeSH Terms])) OR (registries[MeSH Terms]) | 149,319 |
| 6 | 4 OR 5 | 201,949 |
| 7 | survival data[Title/Abstract] OR survival dataset[Title/Abstract] OR survival datasets[Title/Abstract] OR survival analysis[Title/Abstract] OR survival analyses[Title/Abstract] OR time-to-event[Title/Abstract] OR time to event[Title/Abstract] | 25,925 |
| 8 | (analyses, survival[MeSH Terms]) OR (analysis, survival[MeSH Terms]) | 332,374 |
| 9 | 7 OR 8 | 373,468 |
| 10 | **3 AND 6 AND 9** | **98** |

| **DATABASE=EMBASE, SEARCHED ON MARCH 9 2023** | | |
| --- | --- | --- |
| 1 | machine learning.mp. | 124,022 |
| 2 | exp machine learning/ | 385,803 |
| 3 | 1 OR 2 | 398,585 |
| 4 | (electronic health records or electronic health record or electronic medical record or electronic medical records or claims data or claims dataset or claims datasets or administrative claim or administrative claims or disease registries or disease registry or registry data or registry dataset or registry datasets).mp. | 193,867 |
| 5 | exp electronic medical record/ or exp health insurance/ or exp patient registry/ | 455,842 |
| 6 | 4 OR 5 | 533,248 |
| 7 | (survival data or survival dataset or survival datasets or survival analysis or survival analyses or time-to-event or time to event).mp. | 129,561 |
| 8 | exp survival analysis/ | 44,718 |
| 9 | 7 OR 8 | 129,561 |
| 10 | **3 AND 6 AND 9** | **159** |

**Additional Supporting File 1: Part II. Inclusion/Exclusion Criteria for Screening Articles**

*Population:* General population seen in routine care practice (requiring real-world datasets)

*Intervention:* None.

*Comparator:* None.

*Outcomes:* time-to-event clinical outcomes

Timing: From database inception and March 9^th^, 2023

Setting: No limitation.

*Inclusion Criteria:*

(1). must use at least one machine learning model for survival analyses; (2). the risk predictive modelling is based on population-based databases; (3). be an original research paper; (4). full texts in English; (4) abstract, not a full-length article

*Exclusion Criteria:*

(1) Not population-level structured data (e.g., RCT, simulation data, imaging/text data); (2) No ML based modeling (e.g., ML for feature selection); (3) No survival prediction (e.g., binary classification).
