# Supplementary material for: Application of machine learning in predicting survival outcomes involving real-world data: a scoping review
Source: BMC Med Res Methodol. 2023 Nov 13;23:268. doi: 10.1186/s12874-023-02078-1 (PMC10641971; doi:10.1186/s12874-023-02078-1)
Supplement: Supplementary file 2 — Additional file 2: Table S1. Characteristics of Included Studies on ML Predictive Models for Survival Analyses (N=28 studies). Table S2. Performances of Included Studies on Survival Analyses Using ML Algorithms (N=28 Studies). Table S3. Risk of Bias Assessment (N=28 studies). [file 12874_2023_2078_MOESM2_ESM.docx]

**Additional file 2: Part II.Table S1-Table S2**

**Table S1. Characteristics of Included Studies on ML Predictive Models for Survival Analyses (N=28 studies)**

| **Study** | **Data source** | **Study Population** | **Sample Size** | **Outcomes** |
| --- | --- | --- | --- | --- |
| Gandin 2023 | Europe/Italy, Cardiovascular Observatory of Trieste, Electronic health record, November 1, 2009 until December 31, 2018 | T2DM | 10614 | time to HF of advanced diagnosis |
| Kadra-Scalzo 2022 | South London and Maudsley, Electronic health record,1st January 2007 and 31st December 2017 | diagnosis of schizophrenia, schizotypal or delusional disorder | 1515 | Treatment resistant schizophrenia |
| Yan 2022 | *SEER-Medicare 2000 to 2018* | chondrosarcoma | 3145 | survival time |
| Adeoye 2021 | HK Hospital Authority Clinical Management System of the Quee n Mary hospital, Electronic health record, January 1, 2000, and October 1, 2019 | oral cavity cancer | 313 | disease-specific and overall survival |
| *Li 2021* | *2006–2014 in the Veterans Health Administration data* | *follicular lymphoma* | *523* | *survival time* |
| Adeoye 2021 | HK Hospital Authority Clinical Management System of the Quee n Mary hospital, Electronic health record, 1 January 2003, and 31 December 2019 | *follicular lymphoma* | 1098 | malignant transformation |

| **Study** | **Data source** | **Study Population** | **Sample Size** | **Outcomes** |
| --- | --- | --- | --- | --- |
| Lee 2021 | HK public hospital, Electronic health record, 1 January 1997 and 20 June 2020 | Long QT syndrome | 142 | time to ventricular tachycardia/ventricular fibrillation |
| Wongvibulsin 2019 | US Registry data November 2003 and April 2015 | left ventricular ejection fraction | 382 | sudden cardiac arrest |
| Wolfson 2015 | US Registry data November 2003 and April 2017 | diabetes | 87363 | cardiovascular risk |
| Steele 2018 | England based 4 hospitals, Electronic health records | coronary artery disease (myocardial infarction [MI], unstable angina or stable angina) | 82197 | all-cause mortality |
| *She 2020* | *SEER-Medicare 2010 and December 2015* | *NSCLC stage I to III (3119 death)* | *17 322* | *tumor, node, and metastasis stage, lung cancer–specific survival* |
| *Haohui Yu, 2022* | *US Survielance, Epidemiology and EndReults (SEER)* | *patients with rectal adenocarcinoma* | 49275 | *survival rate of patients with rectal adenocarcinoma* |
| Enrico Longato 2021 | the administrative claims of  the Veneto region, in North East Italy | diabetic patients | 214676 | 4P-MACE composite endpoint, i.e., the first occurrence of death, heart failure, myocardial infarction, or stroke |
| *Xin Dai 2022* | *gathering all prostate cancer patients diagnosed between 2001- 2017 in the VA cancer registry* | *prostate cancer patients* | 112276 | *PSA > 50 ng/ml • Metastatic diseases • Prostate cancer mortality.* |

| **Study** | | **Data source** | | **Study Population** | | **Sample Size** | | **Outcomes** |
| --- | --- | --- | --- | --- | --- | --- | --- | --- |
| Yuemin Nan 2022 | | real-world electronic health record (EHR) data from the China Registry of Hepatitis B (CR-HepB) database. | | patients with HBV infection | | 396 | | the time to HBV turning negative, detect HCC |
| Arturo Moncada‑Torres 2022 | | data from the Netherlands Cancer Registry | | *non-metastatic breast cancer patients* | | 36658 | | breast cancer survival |
| Alvin D. Jeffery 2018 | | US electronic health records of adults admitted to a large urban academic medical center from 2006 through 2015. | | adults admitted to a large urban academic medical center from 2006 through 2015 | | 980 | | cardiopulmonary resuscitation |
| Eileen M. Hsich 2020 | | US electronic health records of adults admitted to a large urban academic medical center from 2006 through 2015. | | all adults who underwent isolated heart transplantation | | 30606 | | Post–heart transplant mortality risk |
| Guihong Wan 2022 | | US electronic health records of adults admitted to a large urban academic medical center from 2006 through 2015. | | 1172 from the Mass General Brigham healthcare system (MGB) and 548 from the Dana-Farber Cancer Institute (DFCI). | | 1720 | | *melanoma recurrence* |
| Hugo Loureiro 2021 | | Germany, Flatiron Health (FH) electronic health record (EHR)-derived de-identified database; a nationwide EHRderived de-identified database; the OAK (phase III clinical trial) dataset (external validation) | | pan-cancer populations | | 136000 | | overall survival |
| **Study** | **Data source** | | **Study Population** | | **Sample Size** | | **Outcomes** | |
| Annelaura B Nielsen, 2019 | Danish Registry data for long-term disease histories | | Danish ICU patients | | 230000 | | ICU mortality prediction | |
| Emily Kawaler 2011 | the Marshfield Clinic Research Foundation’s Personalized Medicine Research Project (PMRP) cohort. | | they were over 18 years of age and lived in one of 19 zip codes surrounding the city of Marshfield, Wisconsin, and if at least one member of the household had received care at the Marshfield Clinic within the previous three years. | | 5000 | | risk for post-hospitalization venothromboembolism (VTE) | |
| *Yu Tian 2018* | *2004 to 2013 who were followed up through the end of 2013 were extracted from the Surveillance Epidemiology End Results registry* | | *Patients diagnosed with nonmetastatic colorectal cancer* | | *128061* | | *survival for nonmetastatic colorectal cancer patients.* | |
| Rasmy 2022 | electronic health record data from 87 US health-care systems derived from the Cerner Real-World COVID-19 Q3 Dataset up to September 2020. | | COVID-19 | | 247960 | | (1)in-hospital mortality, (2) need for mechanical ventilation | |
| Gensheimer 2021 | EMR (Epic, Verona, WI) data for patients seen for metastatic cancer in the Stanford Health Care system from 2008–2020. | | metastatic cancer patients | | 14600 | | Survival outcome | |
| Kar 2021 | India based hospitals, a standardized template and electronic medical records of six Apollo Hospital centers (from April to July 2020) | | COVID-19 | | 1393 | | mortality risk | |
| Abdulaal 2020 | electronic health records, a single west London hospital, 2020 | | COVID | | 398 | | death occurring during hospital admission | |
| Rawshani 2019 | Swedish National Diabetes Register, 1998 to 2014 | | patients with type 1 diabetes mellitus | | 32611 | | 1) all-cause mortality ; 2) cardiovascular outcomes (fatal/ nonfatal MI, fatal/nonfatal stroke (henceforth referred to as stroke), and hospitalization for HF.) | |

**Table S2. Performances of Included Studies on Survival Analyses Using ML Algorithms (N=28 Studies)**

| **Study** | **ML Algorithms** | **Model Validation** | **ML model performance*** | **Best ML** | **CPH model performance** | **ML or CPH Better** |
| --- | --- | --- | --- | --- | --- | --- |
| Gandin 2023 | deep neural network survival method (PHNN) | 70% (train), 15% (test) and 15% (validation) | 0.768 | \ | ML > CPH (s 0.734) | ML > CPH (0.734) |
| Kadra-Scalzo 2022 | Cox LASSO | 100-time repeated 10-fold cross-validation | 0.6 | \ | \ | \ |
| Yan 2022 | neural multi-task logistic regression and random survival forest | 7:3 1000-repeated random search with 5-fold crossvalidation | 0.832 (DeepSurv) and 0.821 (NMTLR) | \ | ML > CPH | ML > CPH |
| Adeoye 2021 | DeepSurv, DeepHit, neural net-extended time-dependent cox model (Cox-Time), and random survival forest (RSF) | Five-fold cross-validation was used with 80% training and 20% test | DeepSurv (Overall: 0.74 and disease-specific: 0.85); RSF (Overall: 0.77 and disease-specific: 0.89); Cox-Time (Overall: 0.75 and disease-specific: 0.87); DeepHit (Overall: 0.73 and disease-specific: 0.84) | DeepSurv | \ | \ |
| Li 2021 | random survival forests | Five-fold cross-validation was used with 80% training and 20% test | RSF: 0.73 | \ | CPH (0.74) >ML | CPH (0.74) >ML |
| Adeoye 2021 | Cox-Time, DeepHit, DeepSurv, random survival forest (RSF) | external validation (previously published dataset) | DeepSurv (0.82), RSF (0.73) | DeepSurv | \ | \ |
| Lee 2021 | Random  survival forest | fivefold cross-validation | 0.91 | \ | RSF>CPH (0.82) | RSF>CPH (0.82) |

| **Study** | **ML Algorithms** | **Model Validation** | **ML model performance*** | **Best ML** | **CPH model performance** | **ML or CPH Better** |
| --- | --- | --- | --- | --- | --- | --- |
| Wongvibulsin 2019 | continuous-time, random forest, random survival forest | not reported | AUC (t) | \ | \ | \ |
| Wolfson 2015 | Censored Naive Bayes, Censored Naive Bayes - Principal  Components | 75% train 25% test | CNB: 0.788 CNB-PC: 0.789 | \ | CPH (0.787) but not compare | CPH (0.787) but not compare |
| Steele 2018 | Random survival forests, elastic net model | 2/3 train 1/3 test | RSF: 0.797, elastic net model: 0.801 | \ | CPH not reported | CPH not reported |
| She 2020 | deep learning survival neural network | externally validated | 0.739 | \ | CPH (0.716)<deelp learning survival neural network | CPH (0.716)<deelp learning survival neural network |
| Haohui Yu, 2022 | a seven-layer neural network | training and test cohortsat a ratio of 7:3 | the DeepSurv model yielded a C-index of 0.821. | DeepSurv | ML > CPH | ML > CPH |
| Enrico Longato 2021 | RNN | training: validation: test = 90.6%:4.7%:4.7% | AUROC from 0.812 (C.I.: 0.797 – 0.827) to 0.792 (C.I.: 0.781 – 0.802); C-index from 0.802 (C.I.: 0.788 – 0.816) to 0.770 (C.I.: 0.761 – 0.779). | \ | ML > CPH | ML > CPH |

| **Study** | **ML Algorithms** | **Model Validation** | **ML model performance*** | **Best ML** | **CPH model performance** | **ML or CPH Better** |
| --- | --- | --- | --- | --- | --- | --- |
| Xin Dai 2022 | recurrent deep survival machine, Random Survival Forest (RSF) and Gradient Boosting Machine (GBM) | training (80%) and test set (20%) | RSF=0.85 (0.83), 0.80 (0.83), and 0.76 (0.81), for the 2-, 5-, and 10-year composite (mortality) outcomes, | recurrent deep survival machine | ML > CPH | ML > CPH |
| Yuemin Nan 2022 | Fifteen base predictive models, Random Survival Forest, Adaptive Boosting (ADA) Classifier, The Naive Bayes (NB) | a training (1,675) and test (725) set in the ratio of 7:3. | overall C-index of 0.9075. LGBM presents the highest scores in Acc (0.9548), Pre (0.9511), and F1 (0.9489), | Light Gradient Boosting Machine | \ | \ |
| Arturo Moncada‑Torres 2022 | Random Survival Forests, Survival Support Vector Machines, and Extreme Gradient Boosting [XGB]) | 10-fold cross-validation | XGB even better ( c-index 0.73 ) | XGB | ML > CPH | ML > CPH |
| Alvin D. Jeffery 2018 | logistic regression. random forest and random survival forest | training : validation : test = 50:25:25 | AUROC values of the 4 models ranged from 0.847 to 0.861 | random survival forest | ML > CPH | ML > CPH |
| Eileen M. Hsich 2020 | random survival forests | Not reported | \ | random survival forests | \ | \ |

| **Study** | **ML Algorithms** | **Model Validation** | **ML model performance*** | **Best ML** | **CPH model performance** | **ML or CPH Better** |
| --- | --- | --- | --- | --- | --- | --- |
| Guihong Wan 2022 | GB, RSF, MLP, LR, and SVM, | five-fold cross-validation | a recurrence classification performance of AUC: 0.845 and 0.812; a time-to-event prediction performance of time-dependent AUC: 0.853 and 0.820. | GB and RF | ML > CPH | ML > CPH |
| Hugo Loureiro 2021 | regularized Cox, Random Survival Forests (RSF), Gradient Boosting (GB), DeepSurv (DS), Autoencoder (AE) and Super Learner (SL). | a 90% training and 10% testing dataset (a nationwide EHRderived de-identified database for training and in-sample testing and the OAK (phase III clinical trial) dataset for out-of-sample testing.) | RSF 0.720 [0.716, 0.725], GB 0.722 [0.718, 0.727], DS 0.721 [0.717, 0.726] and lastly, SL 0.723 [0.718, 0.728] | super learner | ML > CPH | ML > CPH |
| Annelaura B Nielsen, 2019 | neural network | a training set (85%) and an independent test set (15%), and a five-fold cross-validation was done during training to avoid overfitting. | Matthews correlation coefficient 0⋅391 for in-hospital mortality | neural network | \ | \ |

| **Study** | **ML Algorithms** | **Model Validation** | **ML model performance*** | **Best ML** | **CPH model performance** | **ML or CPH Better** |
| --- | --- | --- | --- | --- | --- | --- |
| Emily Kawaler 2011 | naïve Bayes, k-nearest neighbor (k-NN) (with filtered variables), support vector machine (SVM), C4.5, and random forest (using REPTree, a form of regression tree) | 10-fold cross-validation methodology | Naïve Bayes, random forest and SVM are among the best learners in this experiment and demonstrate comparable levels of accuracy. | naïve Bayes and random forest | \ | \ |
| Yu Tian 2018 | RSF | a training set (80%) and a test set (20%). | machine learning model was found to be more accurate (0.898[0.895,0.902]) | RSF | ML > CPH | ML > CPH |
| Rasmy 2022 | neural network-based models | external validation | For survival prediction, CovRNN achieved a concordance index of 86·0% (95% CI 85·1–86·9) for in-hospital mortality and 92·6% (92·2–93·0) for mechanical ventilation. | \ | \ | \ |
| Gensheimer 2021 | discrete-time survival model | 80%/20% split | 0.77 (0.73–0.81) | \ | Discrete-Time Survival Model = 0.68 (0.65–0.71) | ML > CPH |
| Kar 2021 | Cox Proportional Hazard Model was used and combined with XGB Algorithm. | Prospective validation cohort was selected of 977 patients (Expired—8.3%) from six centers from July to October 2020 | The validation cohort’s performance is AUC at 0.78, Accuracy score at 0.93, and precision at 0.77. | \ | \ | \ |
| Abdulaal 2020 | ANN | training (80%) and test (20%) sets. | ANN (AUROC=92.6%, 95% CI 91.1–94.1) | \ | Cox regression model (AUROC=86.9%, 95% CI 85.7–88.2) | ML > CPH |
| Rawshani 2019 | random survival forest and gradient boosting | Not reported | Not reported | \ | \ | \ |

**Table S3. Risk of Bias Assessment (N=28 studies)**

| **Study** | **Study Population*** | **Study Attrition†** | **Prognostic Measure‡** | **Outcome Measurement§** | **Study Confoundingǂ** | **Statistical Analysis and Reporting•** | **Quality rating (good, fair, poor)** |
| --- | --- | --- | --- | --- | --- | --- | --- |
| **Orignal Quips** | **The study sample adequately represents the population of interest** | **The study data available(i.e, participants not lost to follow up) adequately represent the study sample** | **The PF is measured in a similar way for all participants** | **The outcome of interest is measured in a similar way for all participants** | **Important potential confounding factors are appropriately accounted for** | **The statistical analysis is appropriate, and all primary outcomes are reported** |  |
| **Tailor to our study** | **Is there an adequate description of study population** | **Did the study provide an adequate description of follow-up information, e.g. describing about any method for handling loss-to-follow-up or deaths?** | **Did the study provide an adequate description of measurement of prognostic factors, e.g. describing about any imputation method for handling missing data?** | **Is there a clear definition of the readmission outcome?** | **Did the study accounted for potential confounding factors from more than three of following domains, such as demographic factors, social determinants of health (SDoH), primary diagnosis or comorbidity index, illness severity, mental health comorbidities, overall health and functional status, prior use of medical services hospitalizations?** | **Did the study conduct any model validation procedure?** |  |
| Gandin 2023 | yes | yes | yes | yes | yes | yes | good |
| Kadra-Scalzo 2022 | yes | yes | yes | yes | yes | yes | good |
| **Yan 2022** | yes | yes | yes (nonparametric missForest imputation method for handling missing data, which imputes missing values based on random forest prediction) | yes | yes | yes | good |
| Adeoye 2021 | yes | yes | yes (Deductive imputation was performed for missing variables in BNI as it  was related to the anatomic sites and T-stage.) | yes | yes | yes | good |
| ***Li 2021*** | yes | yes | yes (RSF handles missing data itself; for the Cox model, missing data were imputed by random forest imputation algorithm [27] using randomForestSRC R package ) | yes | yes | yes | good |
| Adeoye 2021 | yes | yes | Partly, Three features (family history of malignancies, size of the lesion, and lesion border status) had between 64.8% and 94.8% of variables missing and were excluded from further analysis. | yes | yes | yes | fair |
| Lee 2021 | yes | yes | yes | yes | yes | No, did not provide validation methods | fair |
| Wongvibulsin 2019 | yes | yes | yes | yes | yes | no, did not provide validation methods and no predictive performance | poor |
| Wolfson 2015 | yes | yes | yes | yes | yes | yes | good |
| Steele 2018 | yes | yes | yes | yes | yes | yes | good |
| ***She 2020*** | yes | yes | patients were excluded if with any missings in clinical variables | yes | yes | yes | good |
| ***Haohui Yu, 2022*** | yes | yes | not mention any methods in missing value imputation | yes | yes | yes | good |
| Enrico Longato 2021 | yes | yes | not mention any methods in missing value imputation | yes | yes | yes | good |
| ***Xin Dai 2022*** | yes | yes | not mention any methods in missing value imputation | yes | no | yes | fair |
| Yuemin Nan 2022 | yes | yes | not mention any methods in missing value imputation | yes | mainly clinical relevant variables | yes | fair |
| Arturo Moncada‑Torres 2022 | yes | yes | yes (mentioned in previous work) | yes | only age and BC related variables | yes | good |
| Alvin D. Jeffery 2018 | yes | yes | yes | yes | yes | yes | good |
| Eileen M. Hsich 2020 | yes | yes | yes | yes | yes | not mention | fair |
| Guihong Wan 2022 | yes | yes | remove patients if certain variable is missing | yes | yes | yes | good |
| **Hugo Loureiro 2021** | yes | yes | yes | yes | yes | yes | good |
| **Annelaura B Nielsen, 2019** | yes | yes | yes | yes | yes | yes | good |
| Emily Kawaler 2011 | yes | yes | no | yes | yes | yes | fair |
| ***Yu Tian 2018*** | yes | yes | no | yes | yes | yes | fair |
| Rasmy 2022 | yes | no | yes (use readily available structured data from electronic health records in their categorical format without the need for specific feature selection or missing value imputation) | yes | yes | yes | fair |
| Gensheimer 2021 | yes | yes | no | yes | yes | yes | fair |
| Kar 2021 | yes | no (The research team is currently undertaking the analysis of the follow-up care of these patients (survivors).) | no (The initial development cohort was 1435. Forty-two patient’s data were dropped owing to missing fields. No imputations were used in the development or validation cohort) | yes | yes | yes | poor |
| Abdulaal 2020 | yes | yes | yes (There were no missing data in the variables used for analysis.) | yes | yes | yes | good |
| Rawshani 2019 | yes | yes | yes (Missing data were imputed with the Multivariate Imputation by Chained Equations (MICE) methods.) | yes | yes | no | fair |
| * Study describes inclusion criteria for selecting patients, and for enrolled patients describes duration and severity of symptoms, and for enrolled patients describes demographics(at least age), and setings. † Data for hospital readmission outcome available for at least 80% of study populaton at the time of measurement. † Data for hospital readmission outcome available for at least 80% of study populaton at the time of measurement. ‡ Study describes appropriate methods for measuring prognostic factors. § Study describes reproducible and approproate methods to define and identify readmission; transfers and deaths during index hospitalization were excluded. ǂ Study includes at least the following predictors: demographics, length of stay, cormorbidities. • Study includes methods of validation. | | | | | | | |
