# Supplementary material for: Application of machine learning in predicting survival outcomes involving real-world data: a scoping review
Source: BMC Med Res Methodol. 2023 Nov 13;23:268. doi: 10.1186/s12874-023-02078-1 (PMC10641971; doi:10.1186/s12874-023-02078-1)
Supplement: Supplementary file 4 — Additional file 4. [file 12874_2023_2078_MOESM4_ESM.docx]

| **Domain** | **Key items** | **Reported on page #** |
| --- | --- | --- |
| **SOURCE OF DATA** | Source of data (e.g., cohort, case-control, randomized trial participants, or registry data) | PAGE #6 |
| **PARTICIPANTS** | Participant eligibility and recruitment method (e.g., consecutive participants, location, number of centers, setting, inclusion and exclusion criteria) | PAGE #7 |
|  | Participant description | NA |
|  | Details of treatments received, if relevant | NA |
|  | Study dates | PAGE #6 |
| **OUTCOME(S) TO BE PREDICTED** | Definition and method for measurement of outcome | PAGE #6 |
|  | Was the same outcome definition (and method for measurement) used in all patients? | PAGE #10-11 |
|  | Type of outcome (e.g., single or combined endpoints) | PAGE #10 AND Additional File 2: Supporting information Table S1 provides information of types of outcome across included studies. |
|  | Was the outcome assessed without knowledge of the candidate predictors (i.e., blinded)? | NA |
|  | Were candidate predictors part of the outcome (e.g., in panel or consensus diagnosis)? | NA |
|  | Time of outcome occurrence or summary of duration of follow-up | NA |
| **CANDIDATE PREDICTORS**  **(OR INDEX TESTS)** | Number and type of predictors (e.g., demographics, patient history, physical examination, additional testing, disease characteristics) | NA |
|  | Definition and method for measurement of candidate predictors | NA |
|  | Timing of predictor measurement (e.g., at patient presentation, at diagnosis, at treatment initiation) | NA |
|  | Were predictors assessed blinded for outcome, and for each other (if relevant)? | NA |
|  | Handling of predictors in the modelling (e.g., continuous, linear, non-linear transformations or categorised) | NA |
| **SAMPLE SIZE** | Number of participants and number of outcomes/events | PAGE #10 AND  Additional Supporting File 2: Table S1 provides information of sample size by each study. |
|  | Number of outcomes/events in relation to the number of candidate predictors (Events Per Variable) | NA |
| **Domain** | **Key items** | **Reported on page #** |
| **MISSING DATA** | Number of participants with any missing value (include predictors and outcomes) | NA |
|  | Number of participants with missing data for each predictor | NA |
|  | Handling of missing data (e.g., complete-case analysis, imputation, or other methods)  “The most common limitation of these studies was a failure to report how to handle a loss to follow up issues (such as deaths, or other reasons causing the missing values), which is within the domain of study attrition.” | PAGE #9 (Handing of missing data was included in the risk of biasl);  Additional Supporting File 2: Table S3 (Presentation of handling of missing data by each study) |
| **MODEL DEVELOPMENT** | Modelling method (e.g., logistic, survival, neural network, or machine learning techniques) | PAGE #11-12 (Summary of use of ML for all included studies);  AND Table 1 provides ML algorithms across included studies (PAGE #28-30);  Additional Supporting File 2: Table S1 provides the model development for each study. |
|  | Modelling assumptions satisfied | NA |
|  | Method for selection of predictors **for inclusion** in multivariable modelling (e.g., all candidate predictors, pre-selection based on unadjusted association with the outcome) | NA |
|  | Method for selection of predictors **during multivariable modelling** (e.g., full model approach, backward or forward selection) and criteria used (e.g., p-value, Akaike Information Criterion) | NA |
|  | Shrinkage of predictor weights or regression coefficients (e.g., no shrinkage, uniform shrinkage, penalized estimation) | PAGE #14-15 |
| **Domain** | **Key items** | **Reported on page #** |
| **MODEL PERFORMANCE** | Calibration (calibration plot, calibration slope, Hosmer-Lemeshow test) and Discrimination (C-statistic, D-statistic, log-rank) measures with confidence intervals | PAGE #16;  Figure 2 (PAGE #45) presents Boxplot and Beeswarm plot of AUC by ML category;  Table 2 (PAGE #46) presents descriptive statistics of AUC by ML category. |
|  | Classification measures (e.g., sensitivity, specificity, predictive values, net reclassification improvement) and whether a-priori cut points were used | PAGE #10 (Data synthesis and analysis;  PAGE #11-12 (Model Performance); |
| **MODEL EVALUATION** | Method used for testing model performance: development dataset only (random split of data, resampling methods e.g. bootstrap or cross-validation, none) or separate external validation (e.g. temporal, geographical, different setting, different investigators) | PAGE #15;  Table 4 (PAGE #33) provides summary of model validation methods of all included studies; |
|  | In case of poor validation, whether model was adjusted or updated (e.g., intercept recalibrated, predictor effects adjusted, or new predictors added) | NA |
| **Domain** | **Key items** | **Reported on page #** |
| **RESULTS** | Final and other multivariable models (e.g., basic, extended, simplified) presented, including predictor weights or regression coefficients, intercept, baseline survival, model performance measures (with standard errors or confidence intervals) | PAGE #9-12 |
|  | Any alternative presentation of the final prediction models, e.g., sum score, nomogram, score chart, predictions for specific risk subgroups with performance | NA |
|  | Comparison of the distribution of predictors (including missing data) for development and validation datasets | NA |
| **INTERPRETATION AND DISCUSSION** | Interpretation of presented models (confirmatory, i.e., model useful for practice versus exploratory, i.e., more research needed) | PAGE #17-19 |
|  | Comparison with other studies, discussion of generalizability, strengths and limitations. | PAGE #17-19 |

Moons KGM, de Groot JAH, Bouwmeester W, Vergouwe Y, Mallett S, Altman DG, et al. (2014) Critical Appraisal and Data Extraction for Systematic Reviews of Prediction Modelling Studies: The CHARMS Checklist. PLoS Med 11(10): e1001744. <https://doi.org/10.1371/journal.pmed.1001744>

For more information, visit: [**https://methods.cochrane.org/prognosis/tools**](https://methods.cochrane.org/prognosis/tools)
